# Nuclear medicine imaging to guide antibiotic therapy: an expert review

**DOI:** 10.1093/bjr/tqag068

**Published:** 2026-03-26

**Authors:** Domenico Albano, Chiara Lauri, Alberto Signore, Giorgio Treglia

**Affiliations:** Nuclear Medicine, University of Brescia, Brescia 25123, Italy; Nuclear Medicine Department, ASST Spedali Civili di Brescia, Brescia, 25123, Italy; Nuclear Medicine Unit, Department Medical-Surgical Sciences and of Translational Medicine, Faculty of Medicine and Psychology, “Sapienza” University of Rome, Rome 00189, Italy; Nuclear Medicine Unit, Department Medical-Surgical Sciences and of Translational Medicine, Faculty of Medicine and Psychology, “Sapienza” University of Rome, Rome 00189, Italy; Division of Nuclear Medicine, Imaging Institute of Southern Switzerland, Ente Ospedaliero Cantonale, Bellinzona, 6500, Switzerland; Faculty of Biology and Medicine, University of Lausanne, Lausanne 1015, Switzerland; Faculty of Biomedical Sciences, Università della Svizzera Italiana, Lugano 6900, Switzerland

**Keywords:** PET/CT, FDG, SPECT/CT, scintigraphy, antibiotic, nuclear medicine, white blood cells

## Abstract

Bacterial infections and multidrug resistance remain a major global threat. New clinical tools are, therefore, urgently needed to identify bacterial infections and monitor response to antibiotics. Nuclear medicine imaging affords unique opportunities to target and identify bacterial infections, enabling spatial characterization as well as noninvasive, temporal monitoring of the natural course of the disease and response to therapy. In this expert review, we present an overview of the role of [^18^F]-FDG PET/CT and WBC scintigraphy with or without SPECT/CT in predicting treatment response to antibiotics and guide their interruption. A decrease in the [^18^F]FDG uptake or a change of uptake patterns could be potentially used to assess the results of antibiotic therapy in patients with different infectious diseases, despite robust evidence lacking. Concerning WBC scan, their high specificity to target and accumulation over time in infected areas is crucial for the differential diagnosis between infections and sterile inflammation, thus being useful also for therapy follow-up. However, specificity can be reduced in patients with ongoing antibiotics and little is known about the best time window to perform scintigraphy. Finally, we analyzed the potential usefulness of most promising radiolabelled bacterial tracers as potential alternative to FDG and WBC for therapy follow-up.

## Introduction

The breakthrough with penicillin revolutionized medicine, ushering in the golden age of antibiotics and dramatically improving the treatment of bacterial infections.^1^ However, the widespread use of these drugs has unfortunately driven the global surge in antimicrobial resistance (AMR).^2^ By 2050, antimicrobial drug-resistant infections are expected to become the leading cause of death globally and surpass those due to cancer.^3^ This crisis is particularly severe in healthcare, mainly in Intensive Care Units (ICUs), where multidrug-resistant (MDR) pathogens are a major threat. Despite the urgent need, the development of new antibiotics has stalled, leaving current treatments inadequate to effectively combat AMR, which results in significant increases in illness and death.^4^^,^^5^ Effective management of infectious diseases requires swift and accurate identification of bacteria and determination of their antibiotic susceptibility.^6^^,^^7^

Increased mortality rates from infectious diseases are a growing public health concern. Successful management of acute bacterial infections requires early diagnosis and treatment, which are not always easy to achieve. Current diagnostic approaches to detecting bacterial infections and monitoring treatment response, including microscopy, microbiology, and molecular techniques like mass spectrometry and nucleic acid amplification—rely on analyzing clinical samples such as blood, urine, or cerebrospinal fluid to perform cultures, sensitivity testing, and other infection-specific assays. However, it is increasingly recognized that many different infectious foci with distinct bacterial burdens, antimicrobial exposures, and local biology can coexist in the same host.^8–10^ Thus, first first-line tests may not reach a final and correct diagnosis. Furthermore, localized sampling often fails to account for the heterogeneity of infections when multiple lesions are present. These methods provide only a static snapshot, missing the critical temporal shifts that occur as an infection evolves or responds to treatment. Conventional clinical imaging examinations—such as radiography, ultrasound, computed tomography (CT), and magnetic resonance imaging (MRI) are commonly used to detect bacterial infections. However, they are strongly limited because they rely on visualizing anatomical changes. These structural alterations lag behind the initial biochemical events in the infected tissues and are also non-specific, as they represent a mix of the infection and the host’s inflammatory response. So, the early diagnosis may be a challenge due to the difficulty to discriminate with conventional imaging between infectious and inflammatory/neoplastic conditions and within different infectious disease, that may have similar signs and symptoms, especially in patients with chronic infections, in patients with compromised immune systems, and in the elderly.^11^^,^^12^

Molecular hybrid imaging examinations, like positron emission tomography (PET) and single photon emission computed tomography (SPECT), seem to be more appropriate because they can potentially investigate molecular pathways and behaviors of several diseases combining both morphological and functional data. Respectively, white blood cells (WBC) labeled with ^111^In or ^99m^Tc-HMPAO and fluorine-18 fluorodeoxyglucose ([^18^F]-FDG) are the most common radiopharmaceuticals studied in this field with positive findings but several open issues (Table 1).

**Table 1:** Main advantages and limitations of [^18^F]FDG and WBC in infectious diseases.

Radiotracer	Advantages	Limitations
[^18^F]FDG	High sensitivity	Low specificity
	High resolution power	Widespread availability
	Short uptake time	High radiation dose
	Short scan	Need patient preparation (such as fasting)
	Possible quantification (SUV, MTV, TLG)	Affected by recent surgery
WBC	High specificity and sensitivity	Poor resolution power (unless SPECT/CT)
	Not affected by recent surgery	Time consuming

The aim of this article is to review published evidence on the role of nuclear medicine imaging techniques and radiopharmaceuticals in the evaluation of antibiotic treatment.

For this review a comprehensive literature search was conducted across PubMed, Embase, and Scopus using a search strategy based on a combination of MeSH terms and free-text keywords including “Nuclear Medicine,” “PET,” “SPECT,” “scintigraphy,” “antimicrobial,” and “antibiotic.” Authors arbitrarily decided which studies could be included if it specifically addressed the use of radionuclide imaging to evaluate the efficacy of antimicrobial therapy in man. In addition to database searching, the reference lists of relevant review articles were manually screened to identify additional high-impact studies.

## [^18^F]FDG PET

### Mechanism and diagnostic performances of [^18^F]FDG

**Figure 1:**
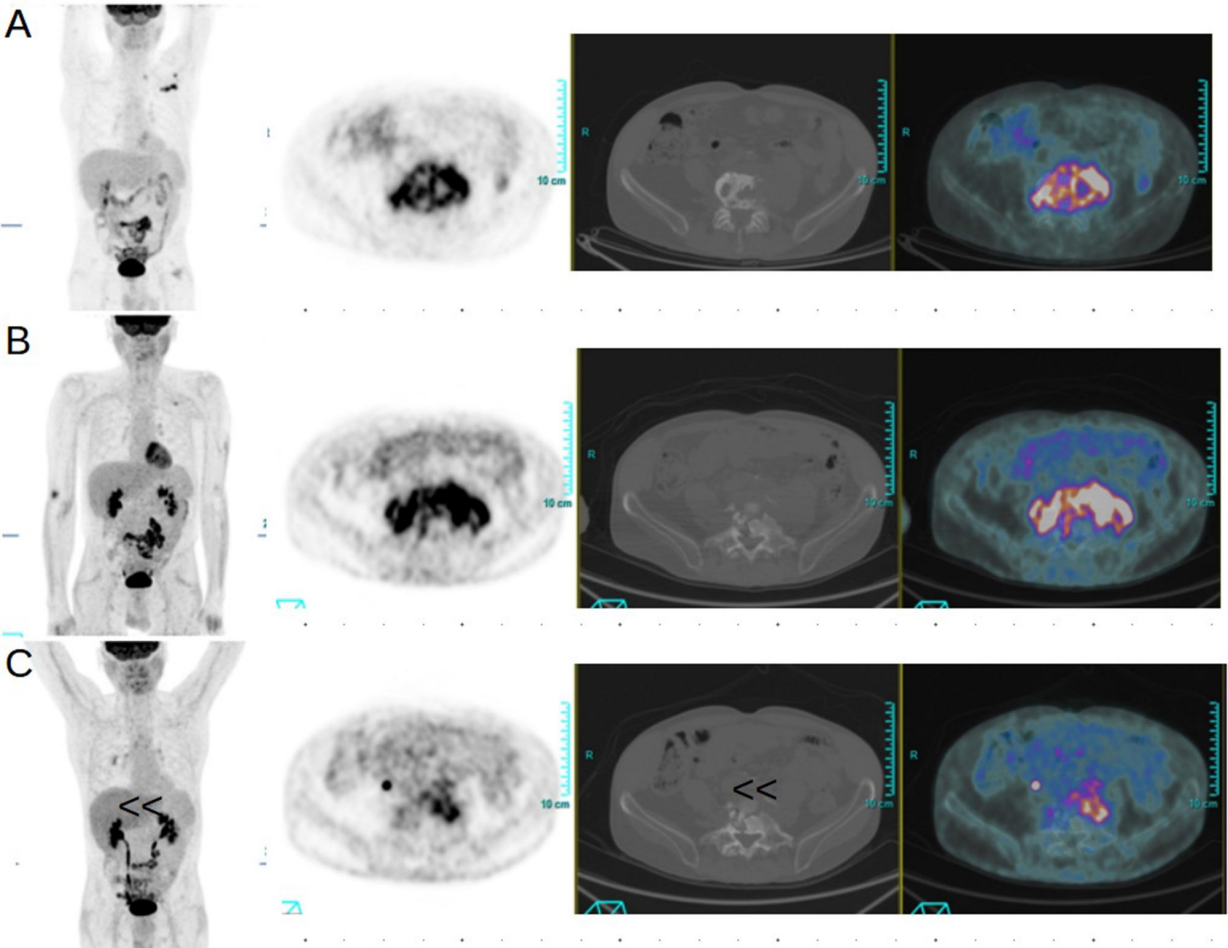
A 60-year-old man with back pain and increased inflammatory markers executed 2-[^18^F]FDG PET/CT showing the presence of an increased uptake in the column corresponding to the fourth and fifth lumbar vertebra. Blood cultures were positive for Staphylococcus aureus. Maximum intensity projection PET (A), axial PET, and fused PET/CT images confirmed the increased 2-[^18^F]FDG uptake in the intervertebral disk and adjacent vertebra suspected for spondylodiscitis. After 3 months of antibiotic treatment, a new 2-[^18^F]FDG PET/CT (B) confirmed the previous uptake increased in extension and intensity demonstrating the lack of effectiveness of therapy. Thus, the patient started a new antibiotic regimen with partial metabolic response demonstrated in the subsequent 2-[^18^F]FDG PET/CT (C) performed after 2 months.

[^18^F]FDG is a radiolabelled glucose analogue taken up by cells via cell membrane glucose transporters and subsequently phosphorylated by hexokinase. The ability of [^18^F]FDG hybrid imaging (PET/CT or PET/MRI) to identify infectious focus is mainly related to the glycolytic activity of the cells involved in the inflammatory response. In particular, it has been demonstrated that cells involved in infection and inflammation, especially neutrophils and the monocyte/macrophage family, are able to express high levels of glucose transporters and hexokinase activity.^13^ Therefore, [^18^F]FDG PET/CT or PET/MRI have been proposed for imaging of several infectious and inflammatory diseases according to evidence-based data^14–17^ and in line with the real-world practice.^18^ Two main topics have been studied about [^18^F]FDG PET and antibiotic/antimicrobial therapy: the possible influence of antibiotic/antimicrobial therapy on [18F]FDG uptake and the use of [18F]FDG PET imaging to evaluate its treatment efficacy, have been both explored **(**Figure 1).

### Diagnostic accuracy

Recently published international nuclear medicine guidelines recommend that [^18^F]FDG PET/CT or PET/MRI in patients with suspected infectious diseases should be preferentially performed prior to the beginning of antibiotic/antimicrobial treatment or as soon as possible thereafter, due to the possible influence of antibiotic/antimicrobial treatment on [^18^F]FDG uptake.^14^^,^^15^ Even if guidelines suggest that [^18^F]FDG PET/CT or PET/MRI studies performed during antibiotic/antimicrobial treatment in patients with suspected infection should be interpreted with caution, a large retrospective study demonstrated that [^18^F]FDG PET imaging correctly identified foci of increased uptake compatible with infection with no false-negative cases in patients receiving antimicrobial therapy.^19^ Based on this study, the administration of antibiotic/antimicrobial therapy seems to have no clinically significant impact on the diagnostic accuracy of [^18^F]FDG PET imaging performed for evaluation of known or suspected infectious diseases.^19^ Overall, literature on this topic is still scarce and larger high-quality studies are needed to verify the impact of antibiotic/antimicrobial therapy and its duration prior to imaging on the diagnostic accuracy of [^18^F]FDG PET imaging.^14^^,^^15^

On the other hand, a decrease in the [^18^F]FDG uptake intensity or a change of [^18^F]FDG uptake patterns could be used to assess the results of antibiotic/antimicrobial therapy in patients with infectious diseases.^14^^,^^15^^,^^20^ Current guidelines recognize that monitoring therapy response remains one of the most important but insufficiently studied potential additional applications of [18F]FDG PET imaging in patients with infectious diseases but appropriate interpretation criteria need to be standardized for several indications. Some examples of recent literature findings for several infectious diseases are reported here below.

### Applications in cardiovascular infections

[^18^F]FDG PET/CT seems to be a promising method for monitoring long-term suppressive antibiotic therapy for infectious endocarditis (IE), assessing its effectiveness, and allowing the identification of a subset of patients in whom antibiotic therapy can be safely discontinued. Nevertheless, the results of a recent international survey conducted to describe current practice patterns of [^18^F]FDG PET/CT use in monitoring long-term suppressive antibiotic therapy for IE, showed a considerable variability in tools and techniques used to monitor patients under therapy, with decisions often made on a case-by-case basis and influenced by the specific type of cardiovascular infection.^21^ Within this context, [^18^F]FDG PET/CT emerged as a valuable tool, contributing significantly to diagnosis, follow-up and the assessment of therapeutic response of IE during long-term suppressive antibiotic therapy. The decrease in [^18^F]FDG uptake values (eg, standardized uptake values—SUV) during follow-up of medical therapy was interpreted by some panelists as indicative of a favorable clinical course, thus supporting the role of [^18^F]FDG for discontinuing antibiotic therapy. The timing of [^18^F]FDG PET imaging during long-term suppressive antibiotic therapy monitoring was debated, with most participants opting for a scan 3-6 months after the baseline evaluation.^21^ Nevertheless, [^18^F]FDG PET/CT application in the management of IE patients receiving long-term suppressive antibiotic therapy remains highly heterogeneous, largely due to the limited availability of robust evidence, the absence of standardized recommendations, and the lack of specific guidelines.^21^

A monocentric retrospective evaluated the usefulness of [^18^F]FDG PET/CT in the follow-up of non-surgically treated prosthetic valve IE not treated with surgery and who had at least 2 [^18^F]FDG PET/CT examinations during their medical management. At the first follow-up [^18^F]FDG PET/CT, 58% of patients showed heterogeneous uptake in the prosthetic valve, thus indicating persistent active endocarditis, and concluding that [^18^F]FDG PET/CT in monitoring of medically treated patients with infective endocarditis provides valuable additional information.^22^ In a separate cohort of 62 patients with non-operated IE, end-of-treatment [^18^F]FDG PET/CT proved to be a powerful prognostic indicator. Nearly half of the participants yielded negative scans, none of whom experienced a relapse over a 10-month median follow-up. While promising, these retrospective results necessitate validation through larger, prospective clinical trials.^23^ Although further prospective studies are needed, follow-up [^18^F]FDG PET/CT also demonstrated its potential role in the management of chronic antibiotic suppression therapy in patients with cardiac implantable electronic device infections when complete device removal is contraindicated.^24^

In selective patients with vascular graft infections who performed several [^18^F]FDG PET/CT during antibiotic treatment, semi-quantitative PET parameters improved over time with associated improvement of laboratory markers of inflammation. These data suggest a potential role of [^18^F]FDG PET/CT not only to detect vascular graft infections, but also to monitor response to antibiotic treatment.^25^

In patients with infective native aortic aneurysms [^18^F]FDG PET/CT proved to be useful in the long-term monitoring of patients as demonstrated by a single-center retrospective cohort study. As compared to the course of C-reactive protein, PET/CT occasionally provided different information, thus modifying therapeutic strategy in infective native aortic aneurysms.^26^

[^18^F]FDG PET/CT could also have an important role in follow-up of infectious foci in *Staphylococcus aureus* bacteremia (SAB). Particularly for complex infectious foci, such as vascular graft infection, [^18^F]FDG PET imaging could be used for treatment monitoring and guiding antibiotic treatment duration. On the other hand, [^18^F]FDG PET may show increased radiopharmaceutical uptake around the graft due to sterile inflammation of the prosthetic material leading to false-positive results and thereby unnecessary continuation of antimicrobial treatment.^27–30^

### Applications in head-neck and spinal infections

There is currently no gold standard for determining when to stop antibiotics in NOE. In a study of 39 patients, [^18^F]FDG PET was evaluated as a tool for therapeutic monitoring. Scans performed 4-9 weeks after starting antibiotics demonstrated high sensitivity for detecting persistent disease; however, the low specificity observed suggests that visual PET markers may stay “positive” even after the infection has been controlled, potentially leading to over-treatment. A complementary quantitative analysis was useful to distinguish between satisfactory and insufficient partial responses.^31^ Another recent retrospective study demonstrated that [^18^F]FDG PET imaging is the first-line imaging modality for evaluating NOE treatment responses, with excellent diagnostic performances even superior to leukocyte scintigraphy in this setting.^32^

According to a retrospective study, [^18^F]FDG PET/CT showed a moderate association with clinical markers used in follow-up of patients with skull base osteomyelitis and is a reliable investigation for assessment of disease status and as a guide along with clinical evaluation for de-escalation of treatment.^33^ C-reactive protein and erythrocyte sedimentation rate had a statistically significant correlation to disease activity at [^18^F]FDG PET/CT in these patients.^34^

Response of pyogenic spine infection to antibiotic therapy is usually based on nonspecific symptoms and inflammation markers. Abnormalities on MRI persist too long to influence therapy decision making. A retrospective study demonstrated that [^18^F]FDG PET/CT could be a timely and robust predictor of successful therapy in spine infections using sequential PET/CT scans done to assess treatment response over a 4-year period and recurrence of infection after stopping treatment as the endpoint. A PET/CT scan with only moderate uptake in the joint with corresponding morphological alterations (like destruction) is associated with a low risk of recurrence. Instead, an unexplained FDG uptake in bone, soft tissue, or spinal canal indicates high risk of recurrence with further antibiotics recommended. Most patients with subtle or localized findings (intermediate risk) did not experience recurrence and stopping therapy could be considered under careful observation.^35^ Another retrospective study on patients with pyogenic spinal infections demonstrated that clinical assessment using clinical symptoms and C-reactive protein for evaluating therapeutic response in these patients is still a useful method in terms of similar recurrence rate compared to [^18^F]FDG PET. However, the high rate of false positives for residual infection can prolong the use of unnecessary antibiotics and overall treatment period.^36^

### Fungal, mycobacterial, and rare infections

The role of [^18^F]FDG PET/CT in assessing treatment response in chronic pulmonary aspergillosis (CPA) was recently assessed in patients with this condition achieving treatment success or failure after 6 months of oral itraconazole. Overall, most PET/CT metabolic parameters improved with treatment; however, [^18^F]FDG PET/CT misclassified one-fifth of the participants.^37^ However, recent data suggest that [^18^F]FDG PET/CT can be of value in monitoring antifungal treatment in invasive fungal infections.^38^^,^^39^

In another study in patients with chronic Q fever caused by *Coxiella burnetiid*, it emerged that the resolution of infection demonstrated by [^18^F]FDG PET/CT can give clinicians reassurance on whether antimicrobial therapy can be ceased earlier, potentially limiting their side effects.^40^

Even if preliminary data seem to suggest a possible role of [^18^F]FDG PET/CT in determining post-treatment response in extrapulmonary tuberculosis, discordance between clinical and PET findings was noticed and the role of PET/CT in this setting needs further evaluation with a larger sample size.^41^^,^^42^

A still unclear point to be further investigated is if the degree of FDG uptake before and after antibiotic therapy may be a surrogate marker of treatment efficacy and could guide the decision of interrupt or de-escalation antibiotics.

### Final remarks

An important prerequisite for optimal use of [^18^F]FDG imaging in patients with infectious diseases including those under antibiotic/antimicrobial therapy remains the presence of a dedicated team with nuclear medicine physicians and infectious diseases specialists. In multidisciplinary meetings [^18^F]FDG PET findings may be discussed with the additional clinical picture of the specific patient leading to new insights and treatment modification.^27^

## White blood cells scintigraphy with or without SPECT/CT

### Diagnostic bases

Given the ability of radiolabelled white blood cells (WBC) to specifically target and accumulate over time in infected areas, they allow an accurate differential diagnosis between infections and sterile inflammation, thus providing crucial information for therapy decision-making.^4^^,^^43^

Nevertheless, specificity can be drastically reduced in patients with ongoing antibiotic therapy and little is known about the best time window to perform WBC scintigraphy after antibiotics withdrawal. Moreover, it is still unclear whether WBC can be used for therapy follow-up and it is strongly suggested to perform WBC scans at least 15 days after therapy withdrawal.

### Applications in musculoskeletal infections

**Figure 2:**
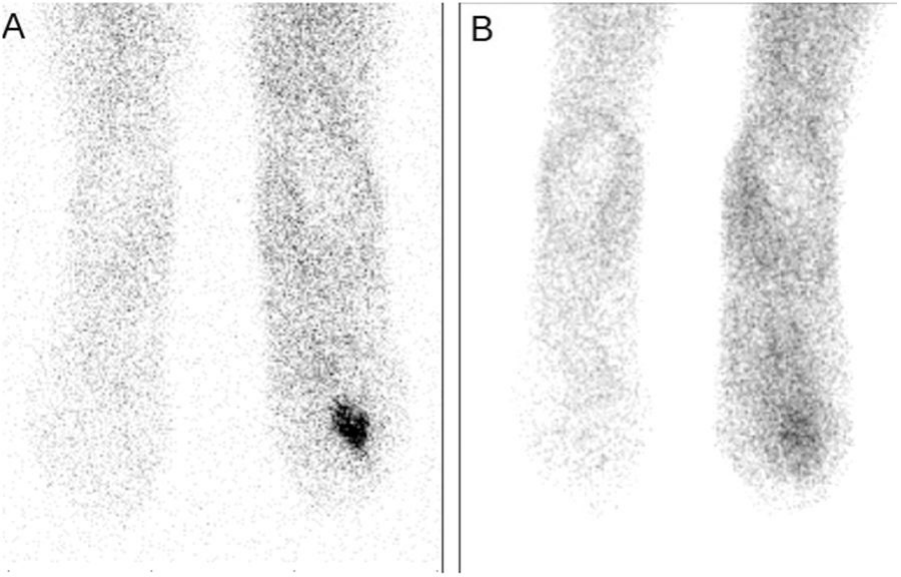
Anterior planar images of WBC scintigraphy in a patient affected by diabetic foot before starting antibiotics (A) and after antibiotics (B) demonstrating a significant reduction of uptake.

Although it is reasonable to apply this imaging modality also to monitor antibiotic treatment response, given its very high negative predictive value (NPV), at the moment, only a few studies addressed this specific topic, in particular, in the field musculoskeletal infections (Figure 2).

In the retrospective paper published in 2018 by Rozenblum-Beddok et al.,^44^ 27 patients with confirmed skull basis osteomyelitis (SBO) were studied with radiolabelled WBC scan (planar images + SPECT/CT) at diagnosis and at the end of antibiotic treatment (3 ± 1 months). The post-therapeutic WBC scan was negative in 26 out of 27 patients, with a significant decrease of target/background (T/B) ratio compared to basal study, and showing a NPV of 88% according to clinical follow-up. Interestingly, patients who experienced disease recurrence after antibiotic suspension had higher T/B ratio at both basal scan and at follow-up scan compared to those who did not recur, thus suggesting the possible prognostic role of semi-quantitative analysis over visual analysis to predict antibiotics treatment efficacy.^44^

### Applications in diabetic foot osteomyelitis

For diabetic foot osteomyelitis (DFO), several authors attempted to assess the possible role of WBC scan in confirming the healing or recurrence after antibiotic therapy.^45–48^ Lazaga et al.^45^ retrospectively evaluated 20 patients studied with sequential WBC scintigraphy (planar + SPECT/CT images) pre- and post-antibiotic therapy to determine treatment success in terms of lack of re-admission within 1 year and wound healing. They reported a sensitivity, specificity, PPV, and NPV of 90%, 56%, 69%, and 83%, respectively.^45^ Vouillarmet et al.,^46^ who compared X-rays, 3-phase bone scan and WBC scan to assess DFO remission at the end of antimicrobial therapy in 29 patients, achieved even better results.^46^ Sensitivity, specificity, PPV, and NPV were 80%, 33%, 20%, and 89% respectively for X-rays, 100%, 12.5%, 15.5%, and 100%, respectively for 3-phase bone scan and 100%, 91.5%, 71.5%, and 100%, respectively, for WBC SPECT/CT scintigraphy. Moreover, all patients who showed a negative WBC scan at the end of antibiotic therapy showed a complete remission during follow-up, whereas 5 out of 7 patients with a persistently positive WBC scan showed a relapse of DFO. Therefore, they concluded that a negative SPECT/CT scan might serve as a tool to detect OM remission and to guide antibiotic therapy.^46^ Optimal duration of antibiotics is still controversial in many clinical conditions, and in particular for diabetic foot infections. Therefore, a few years later, the same group prospectively assessed the ability of WBC scan in predicting DFO remission after 6 or 12 weeks of antibiotic therapy in 45 patients.^47^ In this study, the duration of antimicrobial treatment was defined according to clinical outcome and WBC scan at 6 weeks: if no clinical signs of infection were present and WBC scan was negative, antibiotic therapy was stopped and patients followed up to 1 year. Conversely, antibiotics were continued for further 6 weeks if signs of infections persisted and/or WBC scintigraphy was still positive after 6 weeks of antibiotic therapy. Another WBC-SPECT/CT was performed at 12 weeks. According to clinical and nuclear medicine assessment after 6 weeks, 23 patients underwent a 6-week course of antibiotics and 96% of them were judged in remission after 1 year, the other 22 patients underwent a 12-week course of antibiotics and remission was observed in 73% of cases. Sensitivity, specificity, PPV, and NPV of the 12-week course were 100%, 56%, 46%, and 100%, respectively and it is worth mentioning that also the 6-week course showed very high NPV (96%). Authors concluded that WBC SPECT/CT could be of help to promptly discriminate poorly-responder patients who need a prolonged therapy, from patients that can stop antibiotic treatment, thus reducing side effects, costs and the risk of antibiotic resistance.^46^^,^^47^

Finally, a recent paper by Oz and coll. compared WBC scintigraphy with MRI for diagnosis of DFO and for therapy follow-up purposes. Despite the limitation of this work, it clearly emerged the superiority of WBC scan over MRI at time of diagnosis and the possibility to use the same method at the end of antibiotic treatment to evaluate its efficacy.^48^

### Comparison with [^18^F]-FDG PET/CT

Recently, WBC SPECT/CT was compared to [^18^F]-FDG PET/CT in monitoring treatment response in 17 patients with NOE. The best performance was achieved by comparing basal and post-treatment T/B ratios in terms of SUVmax and using a cut-off of 4.1 (accuracy of 100%) for [^18^F]-FDG PET/CT, and T/B ratios at 4 h SPECT/CT with a threshold 1.9 (accuracy of 88%). The authors concluded that both 18F-FDG PET/CT and WBC SPECT/CT could be used as early prognostic biomarkers to predict therapy response in necrotizing otitis externa.^32^ However, it is not specified in this research if EARL-reconstruction was applied therefore, other studies are warranted to confirm these results.

### Future directions and conclusions

In conclusion, until now WBC-SPECT/CT is not commonly used for follow-up purposes, because solid studies addressing its ability to assess healing after antibiotic therapy in other clinical scenarios are lacking and definite conclusions cannot be drawn.^49^^,^^50^

More large studies are needed to definitively include this approach in the follow-up of medical therapy.

## Other/new radiopharmaceuticals

**Figure 3:**
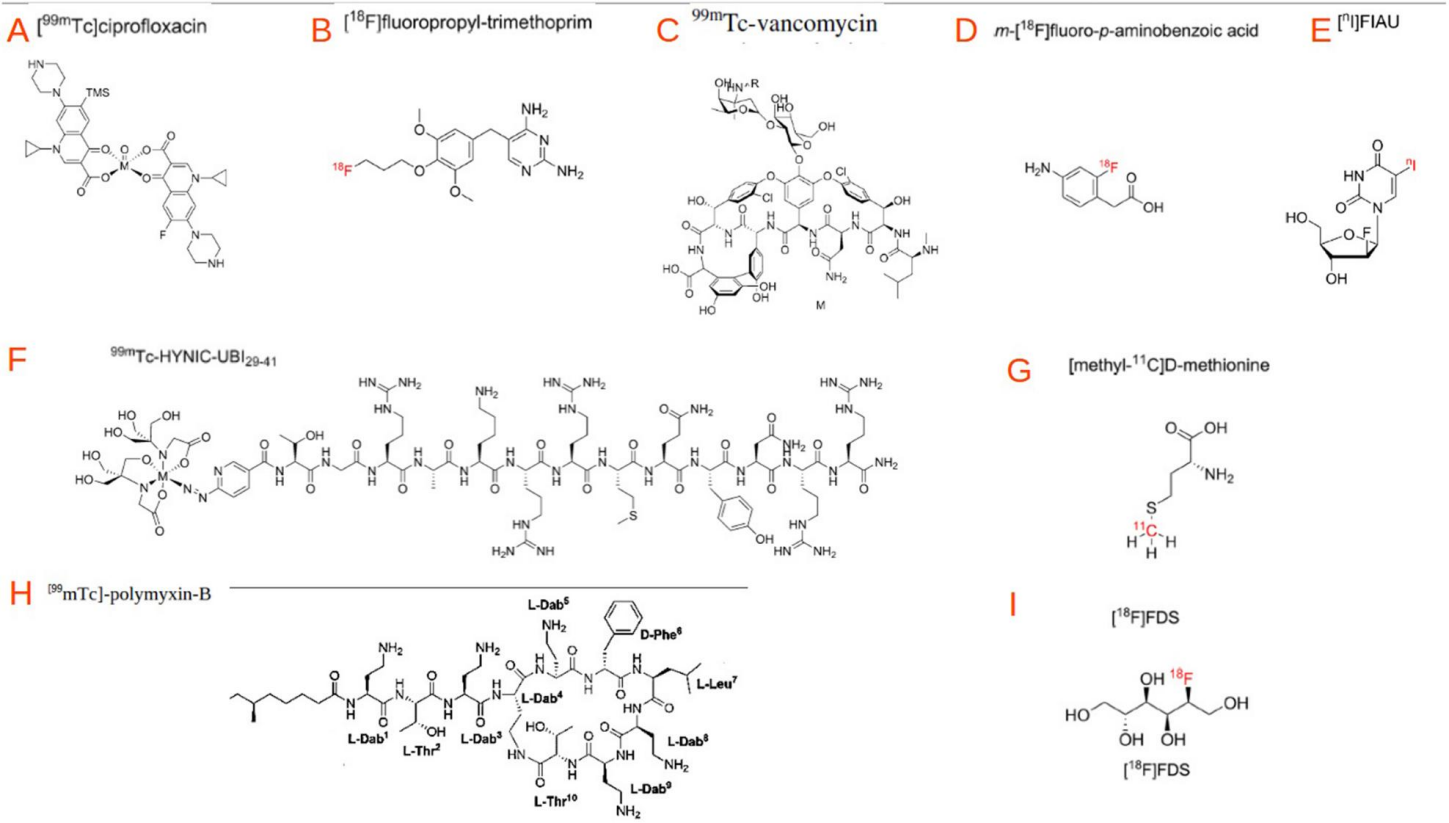
Chemical structures of [^99m^Tc]-ciprofloxacin (A), [^18^F]-FPTMP (B), [^99m^Tc]Vancomycin (C), [^18^F]PABA (D), [124/125I]FIAU (E), [^99m^Tc]-UBI (F), [11C]D-methionine (G), [^99m^Tc]-polymixyn-B (H) [^18F^]-FDS (I).

Beyond [^18^F]FDG and autologous leukocytes labeled with [^111^In] or [^99m^Tc]-HMPAO, other radiopharmaceuticals were investigated, aiming at improving the diagnostic performances and at increasing the ability to detect treatment response in infectious diseases.^7^^,^^51^ Consequently, the practice of directly targeting infectious agents with radiolabeled antibiotics or antimicrobial peptides was introduced, and a number of radiolabeled peptides have been explored.^52^ Nevertheless, one of the main limitations is the lack of translation to clinical practice. Some of these radiopharmaceuticals have targeted unique microbial pathways, including bacteria-specific sugar transport, iron accumulation, cell wall components (like peptidoglycan) (Figure 3). The main aims of these tracers seem to be to identify the type of infections and bacterial species and therefore guide in the choice of the most appropriate antibiotic class. However, this role might have the consequence to help also in the treatment response evaluation. Amongst the wide plethora of these radiopharmaceuticals, only a few of them reached clinical practice, and fewer still have been analyzed for the evaluation of antibiotic response (Table 2). In this part we described the most promising according to our expertise and experience.

**Table 2:** Overview of the main promising radiopharmaceuticals in imaging of bacterial infections.

Tracer	Mechanism	Pathogens	Setting	Specificity	Advantages	Disadvantages
[^99m^Tc]-ciprofloxacin or [^18^F]-ciprofloxacin	Bacterial DNA gyrase, topoismoerase IV enzyme	G+, G−, anaerobic bacteria	Clinical	Low	Targeting drug-sensitive and penicillin-resistant bacteria	Low specificity, broad spectrum
[^18^F]-FPTMP	Inhibition of bacterial dihydrofolate reductase	G+, G−	Preclinical	High	Safety, imaging intracellular bacteria	High background activity, broad spectrum, low sensitivity
[^99m^Tc]-vancomycin	Peptidoglycan precursors	G+	Preclinical	High	Specific for G+, targeting drug-resistance	High hepatic uptake
[^99m^Tc]-polymyxin-B	Binding to membrane lipopolysaccharide	G−	Preclinical	High	Specific for G−	Difficult to label with18F or 11C for PET use
[^99m^Tc]-ubiquidicin	Bacterial membrane	G+, G−, fungi	Clinical	High	Good sensitivity and specificity, therapy monitoring	Broad spectrum
[^99m^Tc]-isoniazid	Blocking fatty acid synthase	Mycobacteria	Clinical	High	High accuracy in muscular infections	Low sensitivity
[^124/125^I]-FIAU	Nucleoside analogue substrate for thymidine kinase	Bacteria	Clinical	Low	Good specificity for selected pathogens	Low sensitivity, high background activity tumor imaging
[^18^F]/[^11^C]-PABA	folic acid biosynthesis pathway	G+, G−	Preclinical and clinical	High	Targeting penicillin-resistant bacteria	Low sensitivity
[^11^C]-D-methionine	Bacterial cell wall	G+, G−	Clinical	High	Good specificity for selected pathogens	Broad spectrum
[^18^F]-fluorosorbitol	Bacterial glucose transporter	G+, G−	Clinical	High	Specific for G−	Uptake in tumors
[^18^F]-maltohexaose, maltotrioses, maltose	Bacterial glucose transporter	Broad spectrum bacteria	Clinical	High	Specific for viable bacteria	Low sensitivity, serum instability

Abbreviations: FIAU = 2'-Fluoro-2'-deoxy-1β-D-arabinofuranosyl-5-iodouracil; FPTMP = fluoropropyl-trimethoprim; G+ = Gram-Positive bacteria; G− = Gram-Negative bacteria; PABA = para-aminobenzoic acid.

### Antibiotics

Fluoroquinolones are inhibitors of bacterial DNA synthesis and bind to the enzyme-DNA interface to arrest replication. This targeted disruption makes them highly effective at stopping bacterial growth at the molecular level.

One of the first agents used in clinical practice for the research of infection foci was [^99m^Tc]-ciprofloxacin. It binds to topoisomerase IV and DNA-gyrase expressed by proliferating bacteria targeting Gram positive, Gram negative, and anaerobic bacteria.^53^

Although it was widely evaluated in hundreds of infected patients with promising results, these initial hopes were dashed when subsequent clinical studies^52–57^ showed poor specificity of [^99m^Tc]-ciprofloxacin for bacterial infections. The main issues of this radiotracer were the low affinity for bacteria (reflected by fast efflux rates from affected tissues) and binding to both mammalian cells and bacteria. Moreover, a recent study speculated that this low diagnostic performance may be associated with the increase of drug-resistance bacteria.^58^

Other radiolabelled natural fluoroquinolone-based antibiotics ([^99m^Tc]Tc-garenoxacin, ^9^[^99m^Tc]Tc-clinafloxacin dithiocarbamate, and [^99m^Tc]TcN-gatifloxacin dithiocarbamate) were tested in mice-models with not robust results.^59–61^

Vancomycin remains the primary therapeutic agent for methicillin-resistant *S. aureus* (MRSA). As a glycopeptide antibiotic, it disrupts cell wall synthesis in Gram-positive bacteria by binding with high affinity to the D-Ala-D-Ala terminus of peptidoglycan precursors. This binding sterically hinders the cross-linking process, leading to an accumulation of precursors and eventual osmotic lysis of the bacterial cell For this reason, [^99m^Tc]-vancomycin was investigated in mice model and ex vivo human tissue demonstrating a good biodistribution with rapid clearance via liver, intestine and kidneys and high tracer uptake in infective sites.^62^ These studies were followed by [^18^F]F-vancomycin that confirmed excellent pre-clinical results.^63^

**Figure 4:**
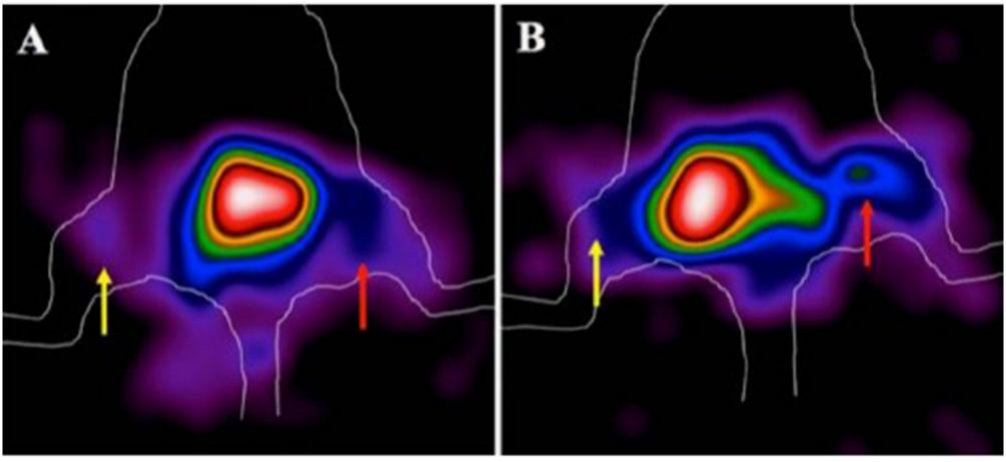
Representative magnified planar γ-camera images of the lower body in mice 6 h post-injection of ^99m^Tc-HYNIC-PMB. Red arrows indicate infection sites inoculated with 10^9^ CFU of *S. aureus* (A) or *P. aeruginosa* (B), while yellow arrows highlight the contralateral control thighs containing only ECM-based hydrogel.

Polymyxin B (PMB) is a cyclic cationic antimicrobial decapeptide used clinically against multidrug-resistant Gram-negative bacteria. Its mechanism relies on binding to lipopolysaccharide (LPS) on the outer membrane, leading to membrane disruption. In nuclear medicine, PMB was conjugated with HYNIC and radiolabelled with ^99m^Tc to develop a SPECT radiopharmaceutical for imaging Gram-negative infections. Radiolabelling produced high efficiency (97%) and stability up to 6 h. In vitro, [^99m^Tc]-HYNIC-PMB showed strong, displaceable binding to Gram-negative strains (*Escherichia coli*, *Pseudomonas aeruginosa*, *Acinetobacter baumannii*), but minimal binding to Gram-positives. In vivo, biodistribution in mice showed predominant renal and hepatic uptake. Targeting studies demonstrated significantly higher target-to-background ratios in Gram-negative infections, with best imaging at 3-6 h post-injection.^64^ These preliminary results support the use of PMB as a promising candidate for non-invasive identification of Gram-negative infections, possibly with PET after conjugation with ^68^Ga (Figure 4).

Another widely investigated SPECT radiopharmaceutical is a fragment of Ubiquidicin (UBI 29-41) radiolabelled with several different isotopes. This is a cationic synthetic fragment derived from the natural cationic AMP ubiquicidin that has been successfully translated into clinical practice due to its ability to discriminate between infection and sterile inflammation, general safety profile, lack of critical side effects^65^^,^^66^ and development of a kit formulations.^67^ Till now, it demonstrated good diagnostic performances in several clinical settings including orthopedic infections^68–70^ and osteomyelitis.^71^^,^^72^ Particularly, [^99m^Tc]-UBI 29-41 seems to be an optimal imaging tool to monitor antibiotic therapy in patients with orthopedic infections^68^; the change of radiotracer uptake 10-14 days after antibiotic treatment may predict responder patients.

More promising findings derived by pre-clinical study on PET tracer [^18^F]F-fluoropropyl-trimethoprim ([^18^F]-FPTMP), analog bacterial dihydrofolate reductase inhibitor. This radiopharmaceutical seems to be able to differentiate between infection, inflammation and tumors in a preclinical model.^73^ The same target radiolabeled with [^11^C]([^11^C]-trimetropim) was tested in humans,^74^ but definitive results are lacking.

### Bacterial metabolism and cell wall

The SPECT radiopharmaceutical [^125^I]I-FIAU or PET radiopharmaceutical [^124^I]I-FIAU have been investigated for the imaging of infections in mice with *E. coli* and *S. aureus*, but in human beings the results are less solid. The main field of investigation were musculoskeletal and orthopedic infections.^75–77^

[^18^F]F-PABA, a substrate for folic acid synthesis in prokaryotes, was also studied for the evaluation of treatment response in mice with methicillin resistant or sensitive *S. aureus* infection. Interestingly, it showed reduced uptake in infected tissues, after oxacillin treatment, that indicates that this tracer can be useful in response monitoring applications. Another radiopharmaceutical related folate synthesis pathway was [methyl-^11^C]-D-methionine. Methionine is a metabolite essential for methylation pathways. This radiotracer was studied especially in muscle infections but the biodistribution was not ideal,^78^^,^^79^ limiting its application in clinical trials.

Other radiopharmaceuticals whose target is carbohydrate metabolism and bacterial glucose transporter are [^18^F]F-fluorosorbitol (FDS) and [^18^F]F-maltohexaose/maltotrioses/maltose. Sorbitol is a substrate that is only metabolized by Enterobacteriaceae, thus it seems logic that [^18^F]-FDS could also be a promising probe capable of differentiating infections with *Klebsiella pneumoniae* or *E. coli* from infections with Gram-positive bacteria ND from sterile inflammations. Moreover, [^18^F]-FDS seems to be useful also for monitoring the effect of antimicrobial interventions.^80^

### Limitations

While this review provides a broad overview, it is limited by its narrative methodology and the somewhat arbitrary selection of featured studies. Additionally, a significant gap remains in clinical practice: despite the diversity of available tracers, there is currently no consensus on a definitive “gold standard” approach for identifying bacterial presence or monitoring antibiotic efficacy.

## Conclusions

This narrative review presents some limitations. Firstly, the narrative and non-systematic nature of the approach may have resulted in the omission of some references on the topics discussed. Additionally, the selection of the most significant studies from the literature was somewhat arbitrary. Despite the availability of many new radiopharmaceuticals, there is no universally accepted approach to the specific detection of bacterial infections and detection of treatment response after antibiotics.

[^18^F]-FDG and ^99m^Tc-WBC are the most investigated radiopharmaceuticals showing promising role in evaluation treatment response, but more robust prospective studies focusing on this aspect are needed to define correct use during antibiotic therapy.

There is also an urgent, unmet clinical need to perform comparative studies between different radiopharmaceuticals having, as a gold standard, the microbiological evaluation of the infection.

The newly developed radiopharmaceuticals hold promising preclinical and clinical data to demonstrate the presence of living pathogens in vivo and to be used for therapy decision-making.
